# Evaluation of Internal and Marginal Accuracy (Trueness and Precision) of Laminates Using DLP Printing and Milling Methods

**DOI:** 10.3390/biomimetics10010067

**Published:** 2025-01-20

**Authors:** Mijun Noh, Habin Lee, Wansun Lee, Jaehong Kim, Jihwan Kim

**Affiliations:** 1 Department of Healthcare Sciences, Faculty of Dental Laboratory Science and Engineering, Korea University, 145 Anam-ro, Seongbuk-gu, Seoul 02841, Republic of Korea; mijune97@korea.ac.kr (M.N.); bin1876@korea.ac.kr (H.L.); 2 Department of Dental Technology, Graduate School, Bucheon University, 25 56th Street, Bucheon 14632, Republic of Korea; ws.lee@bc.ac.kr; 3 Department of Dental Laboratory Science, College of Health Science, Catholic University of Pusan, 57 Oryundae-ro, Geumjeong-gu, Busan 46252, Republic of Korea; kjhong@cup.ac.kr

**Keywords:** 3D printing, accuracy, digital light processing, laminate, zirconia

## Abstract

This study evaluated the internal and marginal accuracy (trueness and precision) of zirconia laminate veneers fabricated using the DLP printing and milling method, employing 3D analysis software program. The maxillary central incisor tooth of a typodont model was prepared by a dentist and scanned using a desktop scanner. An anatomical zirconia laminate was designed using computer-aided design (CAD) software and saved in a standard tessellation language (STL) format. Thirty zirconia laminates were manufactured using a milling machine (MLL group) and a DLP printer (PTL group). All the specimens were scanned, and their internal and marginal areas were edited accordingly. The root-mean-square value was used to assess the accuracy of the internal and marginal areas of the zirconia laminates. Statistical significance was evaluated using the Mann–Whitney U test. Statistically significant differences were found in RMS values for both groups in the internal and marginal areas (*p* < 0.001 and *p* = 0.034, respectively). The MLL and PTL groups differed significantly in terms of precision (*p* = 0.017), but not at the margin (*p* = 0.361). DLP-printed zirconia laminates demonstrated stable and consistent performance, making the technique a reliable option for producing esthetic prostheses.

## 1. Introduction

The increasing interest in esthetic dental restorations has led to an increase in demand for ceramic dental prostheses [1]. Esthetic prostheses are a challenging subject that requires consideration of the individual facial appearance and harmony between the teeth and periodontal tissue. Consequently, various ceramic dental materials with biocompatible and optical properties similar to those of natural teeth have been developed [2]. Among ceramic materials, zirconia is commonly used for dental prostheses for its physical properties, such as biocompatibility and high fracture resistance [3]. These properties have made it a commonly favored option in clinical dentistry, where it is used in a variety of applications, including laminates [4,5] Previous studies have reported that the thickness of anterior zirconia laminates ranges from 0.5 to 0.75 mm [6,7,8]. Pre-sintered blocks by dry milling or fully sintered blocks are mainly used in manufacturing zirconia laminates with a thinner thickness compared to other prosthetic materials [9].

The prosthesis is first designed using a computer-aided design (CAD) program, and the file is exported to a milling machine, which subtracts the zirconia block in the pre-sintered state. Subsequently, high-temperature sintering is performed to produce the final prosthesis. There are some disadvantages of this process, including material loss, increased wear on milling tools, reduced volumetric accuracy due to tool wear, and the risk of undercutting caused by the tool diameter and milling angle [10,11]. On anterior restorations such as laminate veneers, milling thin margins can be difficult and may result in minor irregularities [12,13,14,15] At this point, manufacturing thin and delicate laminates with 3D printing technology can overcome these disadvantages.

3D printing technology involves a slicing process that divides three-dimensional geometry data into a standard tessellation language (STL) format layer by layer, transforms it into multi slice images, and manufactures a shape by stacking materials layer by layer to produce the desired shape [16,17] This approach, in comparison to milling methods, offers the advantage of minimizing material waste, labor, and time while simultaneously expanding design freedom [18]. Additionally, it allows for the creation of complex shapes by stacking various materials in micrometer units [19].

The American Society for Testing and Materials (ASTM) classifies 3D printing technology into seven types as follows: binder jetting, direct energy deposition, powder bed fusion, material extrusion, material jetting, sheet lamination, and vat photopolymerization [20]. Various fields are actively conducting research on lithography-based ceramic manufacturing. In the dental field, vat photopolymerization methods, such as digital light processing (DLP), stereolithography (SLA), and liquid crystal displays (LCD), are being applied clinically, considering their advantages in terms of high resolution, accuracy, printing efficiency, and surface quality [21].

In recent developments, a paste combining zirconia powder with a resin binder has been introduced, enabling the application of zirconia printing using technologies commonly utilized in dentistry. This advancement has facilitated the fabrication of zirconia-based prostheses. Studies have shown that the accuracy of zirconia crowns and bridges manufactured using 3D printing technologies is superior to that of laminates produced by the CAD/CAM method. [22,23,24,25,26].

However, while research on the accuracy of prostheses produced with DLP technology is actively being pursued, the number of studies is limited [27], and research on the accuracy of laminates remains insufficiently elucidated. Research specifically addressing the accuracy of laminates produced by DLP printing is sparse, leaving a critical gap in understanding the potential of this technology in comparison to conventional methods. Thus, this study evaluated and compared the accuracy of the internal and marginal areas of zirconia laminates produced using the milling and DLP printing methods. The null hypothesis was that no difference exists in the accuracy of the internal and marginal areas of zirconia laminates produced by milling and DLP printing.

## 2. Materials and Methods

**Figure 1:**
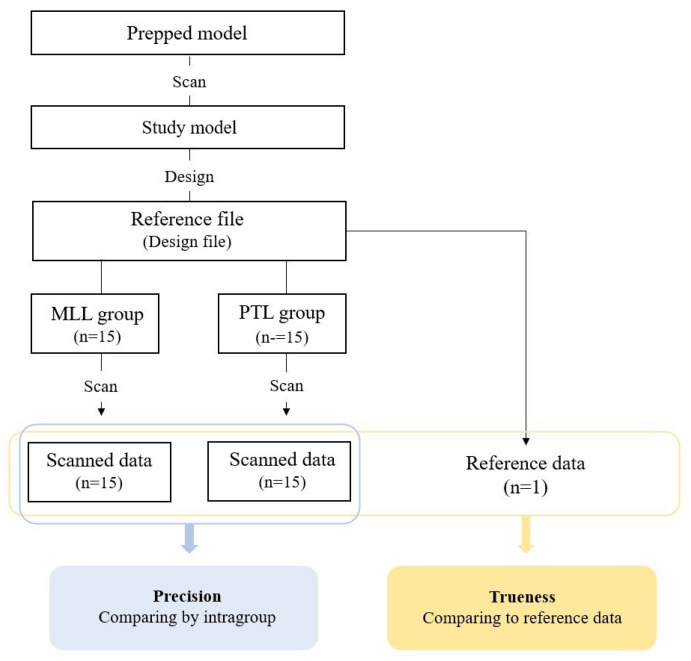
Schematic process of accuracy evaluation.

The overall process of this study is illustrated in Figure 1. A typodont model (D85DP-500B.1, Nissin Dental, Kyoto, Japan) was scanned using a dental scanner (E1, 3Shape, Copenhagen, Denmark). The scanned model was printed using a digital light processing 3D printer (Asiga Max, Asiga, Sydney, Australia), and a dentist prepared the maxillary central incisor tooth in butt joint incisal preparation design to achieve labial reduction of 0.5 mm. The prepared abutment tooth was scanned again, and CAD software (3Shape Dental Designer, 3Shape, Copenhagen, Denmark) was used to design the anatomical zirconia laminate, which was saved in a standard tessellation language (STL) format. It was then exported to CAM software for milling (MLL group) and printing (PTL group). A total number of 30 specimens were prepared, with 15 specimens for each group. The sample size for this study was determined through a power analysis using G*Power software. To achieve a statistical power of 0.80 at a significance level of α = 0.05 for the Mann–Whitney U test, an effect size (d) of 0.8 was assumed, based on a previous study [11].

**Figure 2:**
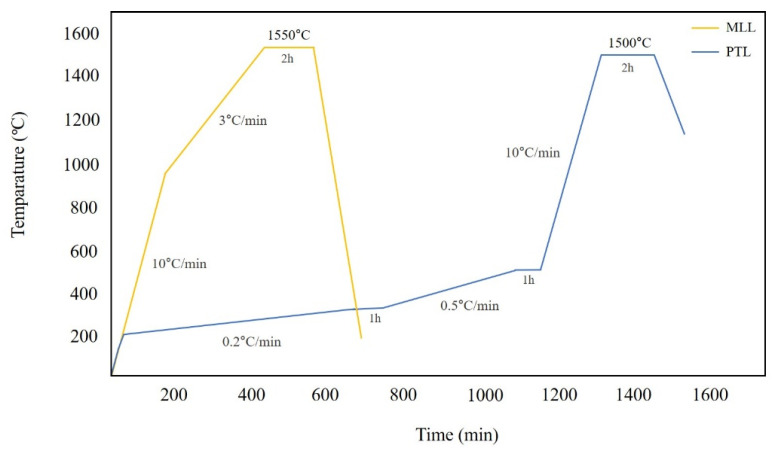
Sintering schedule of MLL and PTL groups.

The toolpath for the STL file was calculated using CAM software (Hyperdent, Follow-me Technology Group, Berlin, Germany) and exported to a milling machine (K5+; Vhf, Ammerbuch, Germany). Subsequently, a pre-sintered zirconia block (Luxen Zirconia 1200 Zr, Dentalmax Co, Seoul, Republic of Korea) was placed, and 15 zirconia laminates were produced. After milling, the supports of the zirconia laminates were removed and sintered in a furnace (Ex-6100, Add-in, Seoul, Republic of Korea) following the recommended schedule from the block manufacturer with a peak temperature set to 1550 degrees (Figure 2). The specimens were finished without additional procedures, such as grinding.

The slicing software (ZIPROS, Aon, Seoul, Korea) of a DLP 3D printer (ZIPRO, Aon, Seoul, Republic of Korea) was used to position the STL files on the build platform. The support was configured following the manufacturer’s guidelines and the output layer height was set at 25 μm, and the STL file underwent a slicing process to generate a multi slice image and subsequently a G code. The 3D printer vat was filled with zirconia paste (ININI-CERA, Aon, Seoul, Republic of Korea) containing a resin binder. Subsequently, the G code was loaded for the commencement of printing

Initially, the platform was lowered, allowing a specific amount of zirconia paste to fill its top surface. After rising, automatic leveling technology was employed, and the zirconia paste was uniformly flattened using the printer’s blade. Ultraviolet (UV) light was projected using a beam projector to solidify one layer at a time. After the build platform descended under the controller command, the zirconia paste refilled the platform’s top surface. The blade flattened it uniformly, and the process of exposing it to UV light was repeated until printing was completed.

The plate attached to the printer was detached, and the output was separated from the plate using a scraper. After cutting the supports from the output, any residual zirconia paste was eliminated using a brush and an isopropanol alcohol solution. The output was then cleaned in an ultrasonic cleaner containing an alcohol solution for 2 min to remove any remaining paste, both internally and externally. Using a sintering machine (Cerafur, Inwhahnc, Seoul, Republic of Korea), the temperature was raised to 500 °C, maintained at increments of 0.2 °C and 0.5 °C for one hour each at room temperature, and then elevated to a peak temperature of 1500 °C for two hours to complete the debinding and sintering processes (Figure 2). The process was completed without additional procedures, such as grinding.

**Figure 3:**
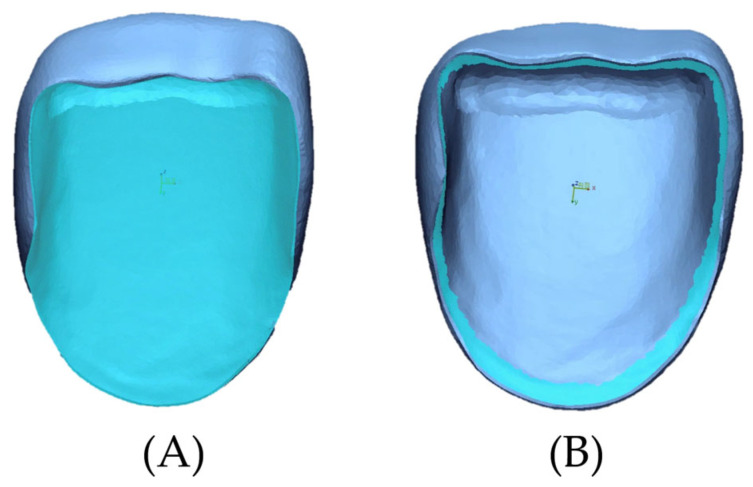
Evaluation area of specimens. (**A**) Internal area. (**B**) Marginal area.

To assess three-dimensional accuracy, a desktop scanner (E4, 3Shape, Copenhagen, Denmark) was used to scan 30 specimens and save them as STL files. The STL files were imported into the 3D evaluation software (Geomagic Control X, 3D Systems, Santa Clara, CA, USA), and to ensure precise alignment, they were segmented based on the internal and marginal areas. Extraneous areas were removed using editing tools, and data were saved. In this process, the marginal area was defined as a margin of 1 mm from the zirconia laminate (Figure 3).

**Figure 4:**
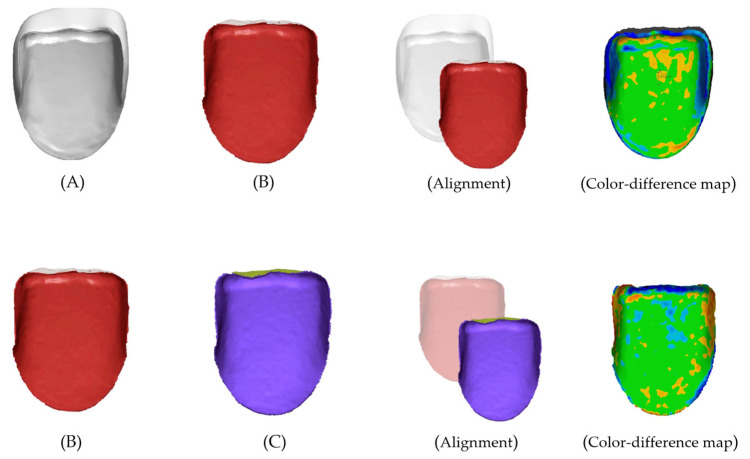
Representative image of 3D alignment. (**A**) Reference design file. (**B**) Scanned file. (**C**) Scanned file. Trueness (**A**,**B**), Precision (**B**,**C**).

For 3D evaluation of trueness, the design file was loaded into the reference data, and the scanned data segmented according to the evaluation area were initially aligned with the reference data (Figure 4). For 3D evaluation of precision, the scanned data were loaded into the reference data, and another scanned data segmented according to the evaluation area were initially aligned with the reference data (Figure 4). A ’best-fit alignment’ was performed to calculate the deviation between the reference and the measured datasets using the root-mean-square (RMS) value. The formula for the RMS value was as follows: RMS = $\frac{1}{\sqrt{n}}\sqrt{\sum_{i=1}^{n}{\left(x_{1,i}-x_{2,i}\right)}^{2}}$

In this formula, n represents the total number of measurement locations, Χ1,i the measurement locations in the reference data, and Χ2,i the measurement locations in the scan data. The RMS value represents the deviation between the different data; the smaller the difference between the reference data and the scanned data, the smaller the RMS value, which indicates accuracy and high three-dimensional agreement. A qualitative evaluation of the 3D deviation between the reference and scanned data was performed using a color-difference map. The maximum deviation was set to +50 μm (red area), and the minimum deviation was set to −50 μm (blue area). The tolerance range was set to ±10 μm (green area).

Statistical analysis was conducted using SPSS version 26 to evaluate the RMS values representing the internal and marginal accuracy of zirconia laminates manufactured by milling and DLP printing methods. The Shapiro–Wilk test was performed to assess the normality of the data for both groups, and the results indicated that the data did not follow a normal distribution (*p* < 0.05). Therefore, the nonparametric Mann–Whitney U test was used for statistical analysis between the two groups. The significance level was set at *p* < 0.05.

## 3. Results

**Figure 5:**
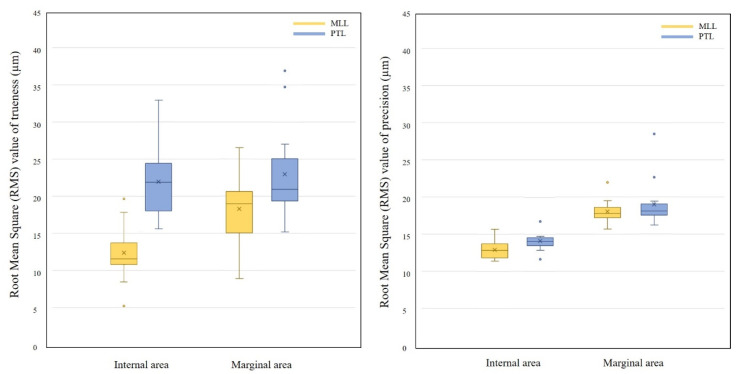
Box plots of the distribution of accuracy RMS values of MLL and PTL groups divided into internal and marginal area: the central line indicates the median, the box represents the interquartile range (IQR), whiskers show the range within 1.5 × IQR, and outliers are marked as dots. “X” denotes the mean values.

The statistical analysis results for trueness and precision are shown in Table 1 and visually represented in the box plots in Figure 5. The box plots illustrate the spread of MLL and PTL data for the internal and marginal evaluation of groups using the following five statistics: minimum, first quartile, median, third quartile, and maximum. Regarding trueness, the MLL group showed the highest trueness with a mean and standard deviation (SD) of 13.87 ± 3.72 µm for the internal RMS value, while the PTL group showed the lowest trueness with 22.97 ± 6.33 µm for the marginal area. RMS values differed significantly between groups in internal and marginal areas (*p* < 0.001 and *p* = 0.034, respectively). Regarding precision, the MLL group had the highest precision with a mean and SD of 12.89 ± 1.31 µm for the internal RMS value, while the PTL group had the lowest precision with 18.97 ± 3.02 µm for the marginal area. However, a statistically significant difference was found between the MLL and PTL groups in terms of precision (*p* = 0.017) but not in margin (*p* = 0.361).

**Figure 6:**
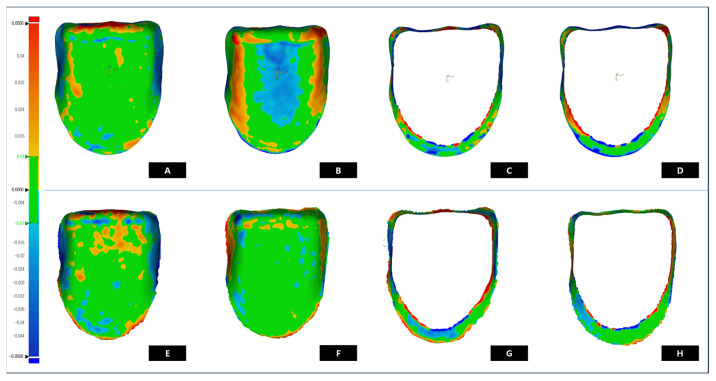
Color-difference maps of trueness and precision deviation of MLL and PTL groups (Trueness: **A**-MLL internal, **B**-PTL internal, **C**-MLL marginal, and **D**-PTL marginal)(Precision: **E**-MLL internal, **F**-PTL internal, **G**-MLL marginal, and **H**-PTL marginal).

The results of the trueness color-difference maps are shown in Figure 6. As shown in Figure 6A, the MLL group revealed a predominantly green area, indicating a discrepancy within a tolerance range of ±10 μm. A blue area was observed in Figure 6C, indicating a negative error. For Figure 6B of the PTL group, a wide blue area was distributed in the center of the internal area, with orange-red positive errors toward the cervical region. As shown in Figure 6D, a blue area was observed at the cervical margin, similar to the internal aspect, a positive red area at the cut, and a predominance of red areas at the cervical margin.

The results of the precision color-difference maps are as follows (Figure 6). As shown in Figure 6E,F, both groups showed a wide range of green areas, indicating a discrepancy within the ±10 μm range. Figure 6E exhibited positive errors in the red area of the incisor region. In addition, Figure 6F was characterized by a red area at the center but showed stable accuracy with a wide green area. Figure 6G,F showed a predominance of negative errors in the cervical region and positive errors at the edge of the margin. Figure 6H showed a positive error and a small red area at the cervical margin compared with Figure 6G. Red or blue errors appear in the marginal area, depending on the characteristics of each processing method.

## 4. Discussion

This study assessed the accuracy of the internal and marginal areas of zirconia laminates produced by DLP printing; further, differences with conventional milling were evaluated. Accuracy encompasses the concepts of trueness and precision and is assessed by measuring these attributes [27]. Trueness indicates the degree of discrepancy between the aligned reference dataset (STL file) and the scanned datasets of the fabricated prosthesis, while precision refers to the degree of consistency among the scanned datasets of the fabricated prosthesis produced using a single manufacturing method. Based on this, the study results showed that when laminates produced by DLP printing were compared to milling, the trueness, meaning processing accuracy, was lower in all measurement areas, and the accuracy, meaning processing reproducibility, was not significantly different from milling only in the marginal area.

Ceramic printing technology makes it relatively easy to produce customized outputs using complex geometries; therefore, attempts have been made to utilize 3D printing methods in dentistry [19,28,29]. In this study, laminate specimens were fabricated using the vat polymerization DLP method. Laminates are mainly manufactured using lithium disilicate and heat-pressing methods or pre-sintered zirconia blocks with CAD/CAM milling [30,31]. The heat-pressing method is completed by creating a wax pattern, attaching sprues, investing, and then removing the investing material after heat-pressing molding [32]. Therefore, a milling method that can be consistently produced with an STL file created by a skilled dental technician was selected as the control group in this study to prevent errors.

Ceramic 3D printing by vat photopolymerization, uses a slurry or paste suspension containing ceramic powders and a photopolymerization polymer binder to enable bonding and photopolymerization between the ceramic powders [33,34,35]. The polymer binder is photocured and polymerized using UV light from the 3D printer, resulting in layer-by-layer polymerization according to the three-dimensional data sliced into two-dimensional images [36,37,38,39]. After washing, a debinding step is performed to remove the resin binder residue, and a sintering step is performed to sinter the green body [40]. Subsequently, a ceramic prosthesis with high density and strength is fabricated. Ongoing research should aim to identify the optimal additive manufacturing technology, materials, and machine settings for demanding production processes.

Zirconia 3D printing using DLP uses a slurry of zirconia powder mixed with a resin-based binder to produce an output. The ceramic particles are bonded and shaped through photopolymerization of the polymer using UV light. The difference in refractive index between the zirconia particles and the photosensitive material affects the scattering effect of UV light. The light-scattering effect from the zirconia particles not only interferes with the photopolymerization required for the green-body output but also expands the curing area corresponding to the curing width. This reduces the penetration depth of the UV light required for curing, resulting in overcuring. The dimensional accuracy of the output could be significantly affected by over-polymerization [41]. This can be reduced by setting the composition of the zirconia slurry and formulating a dispersant with appropriate components to effectively disperse the zirconia powder contained in the photopolymerized resin [42]. Furthermore, because the amount of UV light exposed and the degree of polymerization are proportional, a method for reducing the energy causing the light scattering effect or controlling the size of the zirconia particles can be applied [43].

In addition to the printing process, the volumetric specification of the output is affected by the cleaning of the green body [38,44,45,46]. After printing, an uncured slurry is present on the surface, and various cleaning methods are used to remove it. A solution is used to dissolve the slurry after separating the green body from the platform, and ultrasonic baths have been employed. According to a previous study that measured the geometry, transmission, roughness parameters, and flexural strength of zirconia cleaned using five different post-processing methods, each element was significantly influenced by the cleaning method [47]. The study also highlighted that excessive surface tension occurs owing to slurry residues, leading to the formation of cracks in the green body. Therefore, the post-process cleaning process should be controlled to ensure accurate prosthesis fabrication.

Volumetric shrinkage errors can occur during the debinding process [40]. The green body fabricated layer-by-layer by UV light was in a soft state, without the strength of the sintered zirconia. At this stage, the debinding process was carried out. This caused the binder to combust and decompose, resulting in primary volumetric shrinkage. After debinding, secondary volume shrinkage occurred through sintering, and the grains of the zirconia particles grew and were converted into high-strength zirconia. Excessive volumetric shrinkage appears to be the cause of negative errors in the color difference map of the PTL group in this study. Because of this multiple-volume shrinkage, setting the optimal debinding and sintering temperatures, suspension, and sintering time is essential for producing a prosthesis that aligns with the final specifications set in the CAD software

The inherently complex production of ceramic printing introduces various factors that can impact the 3D accuracy of the final output. In such circumstances, the results of this study can serve as reference data for further research before the parameters are studied. This is because while research on the accuracy of ceramic printing prostheses is actively ongoing, studies on printing laminates are limited, particularly those produced using the DLP printing method. To assess whether a new technology reaches a clinically usable level, continuous accuracy evaluation studies are essential owing to the significant clinical implications of prosthesis accuracy.

The accuracy of the zirconia laminates fabricated using digital technology was assessed based on the degree of agreement between the specifications of the prosthesis, a design file (STL format), and the actual specifications of the fabricated prosthesis. The cement gap between the abutment and prosthesis set by the CAD software is crucial for the fit of the dental prosthesis. If the intended cement gap is not achieved, it may lead to inadequacy, resulting in reduced durability and fracture resistance of the dental prosthesis [48]. Additionally, plaque accumulation caused by an inadequate fit can lead to cement dissolution, contributing to secondary caries, and poses a risk to oral health [49].

Discrepancies are evaluated by performing point-to-point alignments and expressed as RMS values, comparing the RMS values with those of previous studies yields. The average RMS values of the zirconia crowns produced by the DLP printing were approximately 128 μm and 239 μm [50]. The zirconia laminates used in this study had lower mean RMS values, indicating a higher degree of trueness. The average marginal RMS value of the zirconia prosthesis fabricated by SLA printing was 34 ± 5 μm, and this study showed a higher trueness [48]. Compared with previous studies, the internal and marginal areas of the prostheses showed higher or similar accuracy.

A limitation of this study is that only 3-dimensional accuracy of DLP printing was evaluated not considering factors influencing the accuracy of the output in ceramic 3D printing processes. These factors include printing direction, height, ceramic slurry composition, UV light intensity, position and number of supports, temperature and dwell time in postprocessing, order of removing supports affecting geometry of the final output, cleaning time, and changes in the accuracy of final prostheses caused by the variation in shrinkage rates due to sintering protocols of zirconia materials and method [10,36,37,38]. To fully commercialize ceramic printing in dentistry, the findings of this study are essential for accuracy analysis and may serve as a valuable reference for evaluating the accuracy of laminate restorations as materials with improved translucency and color properties are developed. Further studies should be conducted to evaluate the material properties of prostheses printed with translucent materials, evaluate the fit, or examine various printing parameters.

## 5. Conclusions

Within the limitations of this in vitro study, the following conclusion could be drawn. The trueness was lower in all measurement areas for printing, while the accuracy was not significantly different from milling in the marginal area. Within the limitations of this study, this study may contribute to establishing a standard for 3D-printed zirconia laminates, indicating the potential to overcome the processing limitations inherent in milling.

## Figures and Tables

**Table 1:** Mean ± standard deviation (SD), median, 95% confidence interval of difference, and *p*-value of accuracy (trueness, precision) characterized by rms values (µm) of MLL and PTL groups.

Trueness				
Area	Group	Mean ± SD (Median)	95% CI of Difference	*p*
Internal	MLL	13.87 ± 3.72 (13)	11.8–15.93	<0.001
	PTL	21.93 ± 4.67 (21.9)	19.35–24.52	
Marginal	MLL	18.28 ± 4.8 (19)	15.62–20.94	0.034
	PTL	22.97 ± 6.33 (20.9)	19.47–26.48	
Precision				
Area	Group	Mean ± SD (Median)	95% CI of difference	*p*
Internal	MLL	13.87 ± 3.72 (12.83)	12.17–13.62	0.017
	PTL	21.93 ± 4.67 (14.03)	13.32–14.84	
Marginal	MLL	18.28 ± 4.8 (17.77)	17.91–17.77	0.361
	PTL	22.97 ± 6.33 (18.07)	12.17–13.62	

## Data Availability

The raw data supporting the conclusions of this article will be made available by the authors on request.
